# Locoregional recurrence of triple-negative breast cancer with low tumor-infiltrating lymphocytes: a case report

**DOI:** 10.11604/pamj.2025.52.161.50434

**Published:** 2025-12-16

**Authors:** Khadija Khadiri, Zineb Khadrouf, Malak Sadry, Amina Essalihi, Oumaima Bouchra, Majda Taoudi Benchekroun, Mehdi Karkouri

**Affiliations:** 1 Laboratory of Integrative Biology, Faculty of Sciences Ain Chock, Hassan II University of Casablanca, Casablanca, Morocco,; 2 Department of Pathology, Ibn Rochd University Hospital, Casablanca, Morocco,; 3 Laboratory of Cellular and Molecular Pathology, Faculty of Medicine and Pharmacy of Casablanca, Hassan II University of Casablanca, Casablanca, Morocco

**Keywords:** Triple-negative breast cancer, locoregional recurrence, tumor-infiltrating lymphocytes, case report

## Abstract

Triple-negative breast cancer (TNBC) is an aggressive subtype with high histological grade and an increased propensity for early recurrence. Early locoregional recurrence within three months of a Patey mastectomy is uncommon, making this case noteworthy and contributing valuable insight into the biological aggressiveness of TNBC and the prognostic role of tumor-infiltrating lymphocytes (TILs). We report the case of a 49-year-old postmenopausal woman followed for TNBC at the Mohammed VI Oncology Center in Casablanca, Morocco, who presented with a rapidly enlarging inflammatory mass along the outer edge of the left Patey mastectomy scar, extending toward the axillary tail, three months after surgery. Histopathological evaluation confirmed a locoregional recurrence of invasive carcinoma of no special type, revealing a 12.2 cm grade III tumor with a Ki-67 index of 40%, hormone receptor-negative/HER2-negative status, and low TILs (10%). The patient received neoadjuvant chemotherapy, followed by surgical excision of the recurrent lesion, radiotherapy and adjuvant chemotherapy consisting of capecitabine, vinorelbine, and gemcitabine, with good tolerance and no evidence of distant metastasis during follow-up. This case underscores the prognostic impact of low TILs and highlights the importance of considering the tumor microenvironment in risk stratification and personalized management of TNBC.

## Introduction

Breast cancer is the most common malignancy in women. In Morocco, breast cancer is the leading cause of cancer-related mortality among women. Among breast cancer subtypes, invasive carcinoma of no special type of the triple-negative phenotype (TNBC) is distinguished by the lack of hormone receptors, (estrogen receptor (ER) and progesterone receptor (PR)) and human epidermal growth factor receptor 2 (HER2) expression, limiting targeted therapeutic options, although, recently, immunotherapy has been added to the therapeutic options [1,2]. Triple-negative phenotype accounts for more than 35% of breast cancer-related deaths and is associated with higher aggressiveness, increased metastatic potential, and shorter overall survival compared with other subtypes [3]. Tumor-infiltrating lymphocytes (TILs) are recognized as an important prognostic biomarker in breast cancer, particularly in TNBC. High TIL levels are associated with better treatment response and improved survival, whereas low TIL levels may indicate an immunosuppressive tumor microenvironment and accelerated tumor progression [4]. We report here the case of a 49-year-old woman followed for TNBC at the Mohammed VI Oncology Center in Casablanca, Morocco, presenting with locoregional TNBC recurrence after initial Patey mastectomy, with low TIL levels, illustrating the importance of these factors in clinical management. This case is unique because it demonstrates an exceptionally early locoregional recurrence occurring only three months after a Patey mastectomy in a patient with low TILs, highlighting the prognostic significance of the tumor immune microenvironment in TNBC and the need for personalized monitoring and therapeutic strategies.

## Patient and observation

**Patient information:** a 49-year-old postmenopausal, single woman (height: 171 cm; weight: 62 kg; BMI: 21.2 kg/m^2^) followed for TNBC at the Mohammed VI Oncology Center presented with a recurrence of a left breast mass. Menarche occurred at age 13, her medical history was notable for surgery for granulomatous mastitis of tuberculous origin, which was fully resolved, as well as prior left breast cancer treated with a Patey mastectomy, chemotherapy and radiotherapy, with initially favorable outcomes. She had a positive family history of breast cancer in a sister and a cousin. No other chronic illnesses were reported, and her general condition was satisfactory at the time of consultation.

**Clinical findings:** physical examination demonstrated a firm mass arising from the lateral aspect of the left Patey mastectomy scar, extending toward the axillary tail. The lesion was deeply mobile and associated with localized inflammatory signs. No axillary lymphadenopathy was palpated.

**Timeline of current episode:** at initial presentation, the patient was diagnosed with triple-negative breast cancer and underwent a left Patey mastectomy followed by adjuvant chemotherapy and radiotherapy, with a favorable immediate postoperative course. Three months after surgery, she reported the appearance of a new mass along the outer edge of the mastectomy scar, extending toward the axillary tail and associated with local inflammatory signs; clinical examination confirmed a deep, mobile mass, consistent with locoregional recurrence and without evidence of distant metastases. The multidisciplinary team recommended initiating neoadjuvant chemotherapy. After this treatment, reassessment demonstrated a satisfactory tumor response that allowed surgical management. The patient subsequently underwent surgical excision of the recurrent axillary-tail mass. Postoperatively, adjuvant treatment consisted of radiotherapy, followed by additional systemic chemotherapy. Clinical and radiological follow-up showed good treatment tolerance, with no signs of metastatic progression.

**Figure 1:**
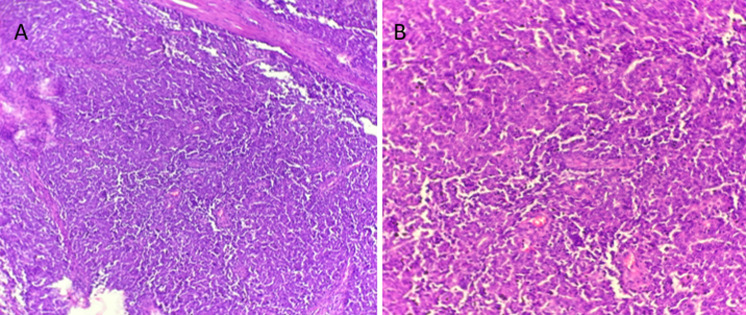
histological sections of invasive breast carcinoma of no special type showing low tumor-infiltrating lymphocytes stained with hematoxylin and eosin; A) X40 original magnification; B) X200 original magnification

**Figure 2:**
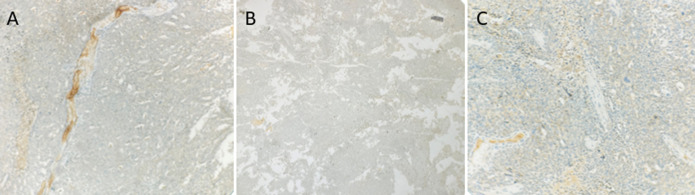
tumor biomarker expression assessed by immunohistochemistry; A) estrogen receptor negative (X100 original magnification); B) progesterone receptor negative (X40 original magnification); C) human epidermal growth factor receptor 2 negative (X100 original magnification)

**Diagnostic assessment:** clinical examination revealed a firm, deeply mobile mass along the lateral aspect of the left mastectomy scar, extending toward the axillary tail. No axillary lymphadenopathy was palpated. Histological examination revealed an invasive carcinoma of no special type (NST); no ductal carcinoma in situ component was identified. The tumor exhibited peritumoral vascular emboli and perineural invasion. Tumor-infiltrating lymphocytes (TILs) were low, estimated at 10%. The deep surgical margin was involved by the tumor. Post-therapeutic changes were present (Figure 1). Immunohistochemical analysis showed negativity for hormone receptors (ER and PR) and HER2 (Figure 2), with a Ki-67 proliferation index of 40%.

**Diagnosis:** based on the combination of clinical, histopathological and immunohistochemical findings, the final diagnosis was locoregional recurrence of triple-negative invasive carcinoma of the left breast.

**Therapeutic interventions:** the patient underwent neoadjuvant chemotherapy, followed by total mastectomy, adjuvant radiotherapy, and subsequent adjuvant chemotherapy in accordance with the institutional protocol. The neoadjuvant regimen consisted of oral capecitabine combined with vinorelbine, while the adjuvant regimen included capecitabine, intravenous vinorelbine, and gemcitabine. The overall treatment was well tolerated, with no clinically significant toxicities observed.

**Follow-up and outcome of interventions:** clinical and radiological follow-up confirmed good treatment tolerance and stable general condition, with no distant metastases.

**Informed consent:** the study protocol was conducted under the ethical principles, the ethical committee of Ibn Rochd University Hospital of Casablanca approved this study under reference 13/2022. The case report is presented anonymously, and all identifying information has been removed to protect patient confidentiality. The patient has expressed her consent to the publication of this report.

## Discussion

This case report provides a detailed clinical, histopathological, and immunohistochemical description of an early locoregional recurrence of triple-negative breast cancer, including the assessment of tumor-infiltrating lymphocytes (TILs). A major strength is the integration of TILs evaluation, which is increasingly recognized as a key prognostic biomarker in TNBC and helps explain the rapid recurrence observed. The report also highlights the unique clinical context of early recurrence only three months after a Patey mastectomy. Another particularity of this patient´s history is her previous episode of mammary tuberculosis, a condition known to generate chronic inflammation. Although a causal link between past granulomatous mastitis and breast cancer progression remains speculative, this background raises interesting questions regarding potential long-term immune modulation and the impact of chronic inflammatory conditions on subsequent tumor biology, especially in immunologically driven cancers such as TNBC. Limitations include its nature as a single case, which limits generalizability, and the lack of longitudinal immune profiling to monitor dynamic changes in the tumor microenvironment. TNBC is well-known for aggressive behavior, with a higher risk of local and distant recurrence compared with other breast cancer subtypes [5]. Tumor-infiltrating lymphocytes (TILs) have emerged as a key biomarker in TNBC, reflecting the host immune response to the tumor. High TILs are consistently associated with improved short- and long-term outcomes; moreover, they serve as a positive predictor of long-term prognosis [6]. Conversely, low TILs are associated with poorer prognosis, decreased chemotherapy response, and accelerated tumor progression [4]. In this context, measuring TILs in the recurrent tumor of our patient contributed meaningful insight into the biology of her relapse, demonstrating that it occurred within an immunologically unfavorable microenvironment. The early recurrence observed in this patient can be attributed to a combination of factors: inherently aggressive TNBC biology, high tumor proliferation, large tumor burden, and an immunosuppressive tumor microenvironment indicated by low TILs. The prior history of mammary tuberculosis, although speculative, may have contributed to chronic immune modulation, potentially influencing tumor-immune interactions. This case emphasizes the scientific rationale for integrating both classical prognostic markers and immune profiling in TNBC management to identify patients at high risk for early recurrence and to guide personalized therapeutic strategies.

## Conclusion

This case highlights the aggressiveness of TNBC with low TILs, explaining the early locoregional recurrence post-mastectomy. Assessment of TILs serves as a key prognostic marker and can guide personalized therapeutic strategies, emphasizing the importance of the tumor immune profile in the management and follow-up of TNBC.

## References

[1] Khadiri K, Khadrouf Z, Mellouki A, Bouchra O, Essalihi A, Naya A (2025). Epidemiological and molecular profile of breast cancer: a retrospective study in Casablanca, Morocco. Pan Afr Med J.

[2] Schmid P, Cortes J, Dent R, Pusztai L, McArthur H, Kümmel S (2022). Event-free Survival with Pembrolizumab in Early Triple-Negative Breast Cancer. N Engl J Med.

[3] Cai SL, Liu JJ, Liu YX, Yu SH, Liu X, Lin XQ (2023). Characteristics of recurrence, predictors for relapse and prognosis of rapid relapse triple-negative breast cancer. Front Oncol.

[4] Obeagu EI, Obeagu GU (2024). Lymphocyte infiltration in breast cancer: a promising prognostic indicator. Medicine (Baltimore).

[5] Toss A, Venturelli M, Civallero M, Piombino C, Domati F, Ficarra G (2022). Predictive factors for relapse in triple-negative breast cancer patients without pathological complete response after neoadjuvant chemotherapy. Front Oncol.

[6] Gao G, Wang Z, Qu X, Zhang Z (2020). Prognostic value of tumor-infiltrating lymphocytes in patients with triple-negative breast cancer: a systematic review and meta-analysis. BMC Cancer.

